# Liver Injury in Patients with COVID-19: A Retrospective Study

**DOI:** 10.7150/ijms.81214

**Published:** 2023-02-05

**Authors:** Yun Feng, Yaping Liu, Qian Zhao, Jiao Zhu, Xiaona Kang, Chen Mi, Peijie Li, Weizhi Li, Guifang Lu, Ai Jia, Shuixiang He, Hongxia Li

**Affiliations:** Department of Gastroenterology, The First Affiliated Hospital of Xi'an Jiao Tong University, Xi'an, People's Republic of China

**Keywords:** COVID-19, liver injury, liver function tests, Severe Acute Respiratory Syndrome Coronavirus 2, Chinese traditional medicine

## Abstract

**Objectives:** The objective of this study is to explore the incidence, characteristics, risk factors, and prognosis of liver injury in patients with COVID-19.

**Methods:** We collected clinical data of 384 cases of COVID-19 and retrospectively analyzed the incidence, characteristics, and risk factors of liver injury of the patients. In addition, we followed the patient two months after discharge.

**Results:** A total of 23.7% of the patients with COVID-19 had liver injury, with higher serum AST (*P* < 0.001), ALT (*P* < 0.001), ALP (*P* = 0.004), GGT (*P* < 0.001), total bilirubin (*P* = 0.002), indirect bilirubin (*P* = 0.025) and direct bilirubin (*P* < 0.001) than the control group. The median serum AST and ALT of COVID-19 patients with liver injury were mildly elevated. Risk factors of liver injury in COVID-19 patients were age (*P* = 0.001), history of liver diseases (*P* = 0.002), alcoholic abuse (*P* = 0.036), body mass index (*P* = 0.037), severity of COVID-19 (*P* < 0.001), C-reactive protein (*P* < 0.001), erythrocyte sedimentation rate (*P* < 0.001), Qing-Fei-Pai-Du-Tang treatment (*P* = 0.032), mechanical ventilation (*P* < 0.001), and ICU admission (*P* < 0.001). Most of the patients (92.3%) with liver injury were treated with hepatoprotective drugs. 95.6% of the patients returned to normal liver function tests at 2 months after discharge.

**Conclusions:** Liver injury was commen in COVID-19 patients with risk factors, most of them have mild elevations in transaminases, and conservative treatment has a good short-term prognosis.

## Introduction

The 2019 novel coronavirus disease (COVID-19) caused by the severe acute respiratory syndrome coronavirus 2 has threatend public health globally. Moreover, rapidly emerging variants of concern have shown greater transmissibility, including alpha (B.1.1.7, UK), beta (B.1.351, South Africa), gamma (B.1.1.28.1, Brazil), Delta (B1.1.617, India), and Omicron (B.1.1.529, South Africa) 1. Among them, Delta is the "fastest and fittest" variant 2. For the past months, we have suffered from the emerging pandemic of Delta and Omicron.

Although COVID-19 patients mainly present with respiratory symptoms, previous reports have shown that patients with COVID-19 are often complicated by liver injury, with the incidence varied from 15% to 53% 3-6. We also found that some COVID-19 patients had liver injury in our work. The causes of liver injury in COVID-19 patients include virus direct attack, cytokine storm, systemic inflammatory response, immune response, hepatic cell ischemia-reperfusion injury, hepatic disseminated intravascular coagulation, hepatotoxicity of drugs, and underlying liver disease, etc. 4, 7-9. Our study retrospectively analyzed the incidence, characteristics, risk factors, and prognosis of liver injury in patients with COVID-19.

## Materials and methods

### Study objects and groups

Our study sample consisted of 384 hospitalized patients of COVID-19 diagnosed between December 9th, 2021 to February 5th, 2022 in Xi'an city, situated in central China. Our inclusion criteria were the presence of the following: positive real-time PCR of severe acute respiratory syndrome coronavirus 2 delta (B1.1.617, India) variants, and our exclusion criteria were the presence of: failure of cooperating with follow-up. All subjects except the deceased received follow-up 2 months after discharge. We broke down the sample into the liver injury group and control group. The liver injury group included COVID-19 patients with abnormal liver function tests above the upper limit of normal during hospitalization. The cases that had normal liver function tests were placed in the control group.

### Clinical Data collection

We collected and analyzed all the clinical and demographic data of the study objects. Demographic data included sex and age. Clinical data of the patient's baseline conditions included history of liver diseases, other underlying diseases, statin use, alcoholic abuse, and body mass index. COVID-19 related clinical data included type of COVID-19, fever, gastrointestinal symptoms, liver function tests, minimum white blood cell, minimum lymphocyte, maximum D-dimer, maximum prothrombin time, maximum activated partial thromboplastin time, maximum C-reactive protein, maximum erythrocyte sedimentation rate during hospitalization, and therapies, such as Chinese traditional medicine, acetaminophen, low molecular weight heparin, COVID-19 neutralizing antibody, hepatoprotective drugs. Mechanical ventilation, intensive care unit (ICU) admission, and death were prognostic indicators. For liver function tests, we mainly focus on maximum serum alanine aminotransferase (ALT), aspartate aminotransferase (AST), alkaline phosphatase (ALP), γ-Glutamyl transferase (GGT), total bilirubin, indirect bilirubin and direct bilirubin during hospitalization.

### Statistical analysis

We used SPSS 24.0 (SPSS Inc., Chicago, IL, USA) for statistical analysis of the data. Categorical variables were expressed as the number of cases (percentage) and analyzed by Pearson χ2 tests between groups. Continuous variables conformed to normal distribution were represented by mean ± standard deviation and analyzed by Student's *t*-tests and ANOVA tests. Continuous variables didn't conform to normal distribution were presented as median (interquartile range) and analyzed by Mann-Whitney *U-*tests. *P* < 0.05 means significant statistical difference.

## Results

### Risk Factors of Liver Injury in Baseline Conditions of COVID-19 Patients

Our study enrolled a total of 384 patients with COVID-19. Table 1 shows the baseline conditions of COVID-19 patients, and patients in the liver injury group and the control group. The patients' average age was 40.6 ± 19.5 years (0 to 87 years). The ratio of male to female patients was 1:1.08. Only 27 (7.0%) patients had history of liver diseases, including nonalcoholic fatty liver disease (3.4%), alcoholic fatty liver disease (1.8%), chronic hepatitis B (1.6%), chronic viral hepatitis C (0.3%), and liver cirrhosis (0.3%). Seven patients (1.8%) were found to have a history of alcohol abuse, meeting the diagnostic criteria for alcoholic liver disease. As many as 68 (17.7%) patients had history of cardiovascular diseases, including hypertension (8.1%), coronary heart disease (6.3%), peripheral atherosclerosis (3.4%), arrhythmia (2.9%), congenital heart disease (0.3%), rheumatic heart disease (0.3%). Thirty-nine patients (10.2%) had hyperlipidemia. Among the patients with cardiovascular diseases or hyperlipidemia, 25 (6.5%) patients had a history of statin use, which may lead to liver injury. We also calculated the patients' body mass index. Some patients were overweight (18.0%) and obese (5.7%), which may be related to nonalcoholic fatty liver disease, hyperlipidemia, hypertension, and diabetes.

COVID-19 patients with liver injury were older than those without liver injury (46.7±18.2 vs 38.7±19.5, *P* = 0.001). Furthermore, COVID-19 patients with liver injury were more likely to have a history of liver diseases (14.3% vs 4.8%, *P* = 0.002), alcoholic abuse (4.4% vs 0.9%, *P* = 0.036), and overweight and obese (28.6% vs 16.4%, *P* = 0.037) compared with the patients without liver injury (Table 1). Sex, history of cardiovascular diseases, hyperlipidemia, and statin use were not statistically different between the liver injury group and control group. Therefore, Age, history of liver diseases, alcoholic abuse, and overweight and obese were risk factors of liver injury in patients with COVID-19.

### Liver Function Tests of Liver Injury in COVID-19 Patients

We included all COVID-19 patients with abnormal liver function tests during hospitalization as patients with liver injury. The median AST (63 (55) vs 19 (13), *P* < 0.001), ALT (68 (62) vs 21 (13), *P* < 0.001) of COVID-19 patients with liver injury were significantly higher than those of the control group, with the median higher than the upper limit of the reference range, but not more than twice the upper limit (Table 1). The median GGT (31 (26) vs 16 (12), *P* < 0.001), ALP (67 (51) vs 58 (49), *P* = 0.004), total bilirubin (13.1 (10.5) vs 13.1 (9.2), *P* = 0.002), indirect bilirubin (9.9 (7.8) vs 10.0 (7.1), *P* = 0.025), and direct bilirubin (3.5 (2.4) vs 2.4 (1.6), *P* < 0.001) of COVID-19 patients with liver injury also significantly increased than those of the control group, still within the reference range.

### Correlation Between Liver Injury and the Severity of COVID-19

Most of the COVID-19 patients were common type (54.2%) and mild type (43.8%) (Table 1). Severe type and critical type of COVID-19 accounted for only 2.1% of all the study objects. Severe and critical type of COVID-19 were more likely to complicated with liver injury (*P* < 0.001). 8.8% of the COVID-19 patients with liver injury were severe or critical type. However, none of the COVID-19 patients without liver injury were severe or critical type.

### Risk Factors of Liver Injury in Clinical characteristics Related to COVID-19

As shown in Table 1, fever was a common symptom in 45.1% of patients. In addition, 32.8% of patients had gastrointestinal symptoms, including anorexia, abdominal discomfort, abdominal pain, bloating, nausea, vomiting, acid reflux, diarrhea, and constipation, etc. Fever and gastrointestinal symptoms did not differ in COVID-19 patients liver injury or not.

The median minimum WBC during hospitalization in COVID-19 patients with liver injury was 3.48×10^9^/L, which was slightly below the lower limit of the normal reference range. However, we found no statistical difference in WBC between the liver injury group and control group. The lymphocytes, D-dimer, prothrombin time, and activated partial thromboplastin time were similar in both groups. The C-reactive protein of the COVID-19 patients were higher than the upper limit of the normal reference value, as well as the erythrocyte sedimentation rate of the patients with liver injury. Moreover, patients with liver injury showed significantly higher C-reactive protein (14.2 (10.8) vs 9.3 (4.5), *P* < 0.001) and erythrocyte sedimentation rate (21 (17) vs 17 (10), *P* < 0.001) compared with the patients without liver injury. Results show liver damage correlates with severity of inflammation in patients Therefore, comparison of laboratory tests between the two groups showed that liver injury correlated with the severity of inflammation in COVID-19 patients.

### Correlation Between Liver Injury and Treatment of COVID-19

Up to 88.8% of the COVID-19 patients received Chinese traditional medicine treatment, including Lian-Hua-Qing-Wen (88.8%), Qing-Fei-Pai-Du-Tang (75.0%), Huo-Xiang-Zheng-Qi (18.5%), Xuan-Fei-Bai-Du-Tang (7.0%), Shen-Qi-Shi-Yi-Wei (3.6%), Qiang-Li-Pi-Pa (8.6%), Su-Huang-Zhi-Ke (6.0%), Lin-Yang-Jiao (14.3%), and Others (22.1%) (Table 1). 74.2% of the COVID-19 patients received low molecular weight heparin treatment. Some patients with fever took acetaminophen (32.8%). Neutralizing antibody therapy was used in only a very small number of patients (2.9%).

We found that patients who took Qing-Fei-Pai-Du-Tang orally were more likely to have liver injury than those who did not take it (76 (83.5%) vs 212 (72.4%), *P* = 0.032). There was no statistical difference between the patients with liver injury and those without liver injury whether or not to use Chinese traditional medicine, other Chinese traditional medicines except Qing-Fei-Pai-Du-Tang, low molecular weight heparin, and acetaminophen.

### Correlation Between Liver Injury and Clinical Outcomes of COVID-19

Of all our study subjects, 8 (2.1%) patients were admitted to the ICU and received mechanical ventilation. We found that all the 8 patients had liver injury. Mechanical ventilation (8 (8.8%) vs 0 (0.0%), *P* < 0.001) and ICU admission (8 (8.8%) vs 0 (0.0%), *P* < 0.001) were more common in patients with liver injury than controls (Table 1). One patient (0.3%) died from respiratory failure, chronic obstructive pulmonary disease, and acute coronary syndrome. There was no significant difference in mortality between patients with and without liver injury. The length of hospital stay was similar between the two groups.

### Treatment and Short-term Prognosis of Liver Injury in COVID-19 Patients

All patients with liver injury were discontinued from statins and Chinese traditional medicine. Most of the patients (92.3%) with liver injury were treated with hepatoprotective drugs, significantly higher than the control group (84 (92.3%) vs 0 (0.0%), *P* < 0.001). Hepatoprotective drugs included compound glycyrrhizin, diammonium glycyrrhizinate, polyene phosphatidyl choline, silymarin, bicyclol, reduced glutathione, Ademetionine1,4-Butanedisulfonate, etc. We found that 35.2% (32/91) of the COVID-19 patients with liver injury returned to normal liver function tests before discharge.

Patients whose liver function tests had not returned to normal at the time of discharge needed to continue taking hepatoprotective drugs. In addition, patients with a history of liver disease also received treatment tailored to the cause, including antiviral drugs, a low-fat diet, exercise, and abstinence from alcohol. We also instructed patients to review liver function tests 2 weeks and 2 months after discharge.

The follow-up results showed that 83.5% (76/91) of the COVID-19 patients with liver injury during hospitalization had normal liver function tests at 2 weeks after discharge, and a total of 95.6% (87/91) of them returned to normal at 2 months after discharge.

The remaining four patients with abnormal liver function tests at discharge all had a history of liver disease, including liver cirrhosis, hepatitis C, and alcoholic liver disease. The median total number of days patients took hepatoprotective drugs was 19, from hospitalization to 2 months after discharge.

## Discussion

We found a high risk of liver injury for patients with COVID-19 from our retrospective study. Up to 23.7% of COVID-19 patients had liver injury. Liver function tests in COVID-19 patients with liver injury were characterized by mildly elevated serum AST and ALT. GGT, ALP, total bilirubin, indirect bilirubin, and direct bilirubin were also higher in patients with liver injury. COVID-19 patients with older age, history of liver diseases, alcoholic abuse, overweight and obese were more likely to have liver injury. liver injury also associated with severe and critical COVID-19, increased C-reactive protein, elevated erythrocyte sedimentation rate, mechanical ventilation, ICU admission, and Qing-Fei-Pai-Du-Tang treatment. The prognosis of COVID-19 complicated with liver injury was generally good, with 95.6% of patients returning to normal liver function tests 2 months after discharge.

Clinicians should pay attention to monitoring the liver function tests for timely detection and appropriate treatment, since more than 1/5 of COVID-19 patients had liver injury. The incidence of liver injury in COVID-19 patients caused by Delta variant infection in our study was consistent with previous reports 3-6. Most patients with COVID-19 complicated with liver injury showed mildly elevated transaminases, and some patients had elevated GGT, ALP, and total bilirubin, and decreased ALB. Approximately 60% of the COVID-19 patients with liver injury had slightly elevated AST and ALT, with the values between 1-2 times the upper limit of normal 10. Elevated GGT, ALP, and total bilirubin, and decreased albumin were also observed in some COVID-19 patients 10, 11. Total bilirubin was normal or mildly elevated in most COVID-19 patients 10.

Moreover, we draw lessons from our study to notice the liver function tests particularly in patients with risk factors including old age, history of liver diseases, alcoholic abuse, overweight and obese, severe and critical type of COVID-19, mechanical ventilation, ICU admission, high inflammatory indexes, and Qing-Fei-Pai-Du-Tang treatment. Drugs that may lead to liver injury should be avoided in patients with risk factors of liver injury.

Multiple factors make elderly patients with COVID-19 more likely to have liver injury. According to previous reports, elderly patients are at higher risk of developing severe and critical COVID-19 than younger patient 12, 13, and are more likely to use statins and other drugs that may cause liver injury due to underlying cardiovascular and cerebrovascular diseases, hypertension, diabetes, hyperlipidemia, etc. 14. It is also obvious that COVID-19 patients with underlying liver disease are more likely to have abnormal liver function tests, which has been frequently mentioned 15. In addition, elderly patients with a history of liver disease may have a longer course of disease, more severe disease, worse liver function, and poorer prognosis 16. It is also well understood that alcohol abuse is a risk factor for liver injury in patients with COVID-19, since it's the cause of alcoholic liver disease. Studies have reported that alcohol related liver disease patients are particularly vulnerable to severe acute respiratory syndrome coronavirus 2 infection and had worse prognosis 17. Overweight and obese are also risk factors for severe and critical COVID-19, and are common components of metabolic syndrome with nonalcoholic fatty liver disease 12, 13.

In addition to these underlying risk factors, liver injury is closely related to the severity of the COVID-19 disease itself. Meta-analyses have found that COVID-19 patients with acute liver injury had higher odds risk of suffering from severe disease compared with those without acute liver injury 18, 19. Severe and critical type of COVID-19 were associated with high levels of inflammatory indexes, mechanical ventilation, and ICU admission 4, 5, 20. Therefore, high C-reactive protein and erythrocyte sedimentation rate, mechanical ventilation, and ICU admission are also risk factors for liver injury in patients with COVID-19. These risk factors are associated with the pathogenesis of liver injury complicated in COVID-19, including viral attack, systemic inflammatory response, cytokine storm, coagulation dysfunction, endothelial damage, and ischemia-reperfusion injury 4, 7-9.

Drug-induced liver injury is also one of the non-negligible causes of liver injury in COVID-19 21. Common drugs that cause liver injury in COVID-19 patients include lopinavir, ritonavir, oseltamivir, antipyretic drugs, and some Chinese traditional patent medicines 21, 22. Qing-Fei-Pai-Du-Tang, a Chinese traditional medicine formula that including 21 herbs, has proven its effectiveness in mild and common types of COVID-19 23, 24. No other studies have shown that Qing-Fei-Pai-Du-Tang treatment increases the risk of liver injury in COVID-19 patients. Therefore, the risk of liver injury caused by Qing-Fei-Pai-Du-Tang may require further large-sample cohort studies for to explore. The application of Qing-Fei-Pai-Du-Tang in the treatment of COVID-19 may need further optimization and normalization of the course, dosage, or compatibility, etc.

Most COVID-19 patients with liver injury were treated conservatively, including hepatoprotective drugs, and discontinuation of Chinese traditional medicine and statins, and achieved good curative effects and short-term prognosis. Patients with underlying liver diseases also require treatment for both causes and complications. Previous studies have also reported that patients with COVID-19 complicated with liver injury have a good prognosis, expect those with underlying liver diseases 25. COVID-19 patients with chronic liver disease had an increased risk of mortality (risk ratio 2.8), in which liver cirrhosis had the highest risk of mortality (risk ratio 4.6) 26. Alcoholic liver disease and hepatocellular carcinoma are also associated with high mortality in COVID-19 patients 26-28. Chronic viral hepatitis and nonalcoholic fatty liver disease have also been shown to be negative prognostic factors in COVID-19 patients 29, 30. Although the short-term prognosis of patients with liver injury in COVID-19 is good, it is still unclear whether liver injury complicated in COVID-19 has long-term effects on the liver.

The primary advantage of our study is that it is a large cohort study of liver injury in COVID-19 patients caused by Delta variant infection. Moreover, our study comprehensively analyzed the characteristics of liver function tests and risk factors of liver injury. Furthermore, we performed short-term follow-up and prognostic analysis of patients. There are also some limitations to the study. It's a single-center retrospective study. Most of the COVID-19 patients of mild and common types received Chinese traditional medicine-based comprehensive treatment. We have not performed long-term follow-up of the patients.

## Conclusions

In summary, we find that liver injury was common in COVID-19, characterized by mild AST and ALT elevations that respond well to medication. We should pay attention to the monitoring of liver biochemical indexes in COVID-19 patients with risk factors for liver injury, and try to avoid the use of drugs that may cause liver injury.

## Figures and Tables

**Table 1:** Liver Injury of COVID-19 Patients, mean ± SD, median (interquartile range), or N(%).

Characteristic	COVID-19 patients (n=384)	Liver injury group (n=91)	Control group (n=293)	*P* *
Sex				0.604
Male	185 (48.2%)	46 (50.5%)	139 (47.4%)	
Female	199 (51.8%)	45 (49.5%)	154 (52.6%)	
Age, years	40.6±19.4	46.7±18.2	38.7±19.5	0.001
Liver diseases	27 (7.0%)	13 (14.3%)	14 (4.8%)	0.002
Cardiovascular diseases	68 (17.7%)	18 (19.8%)	50 (17.1%)	0.553
Hyperlipidemia	39 (10.2%)	10 (11.0%)	29 (9.9%)	0.763
Statin use	25 (6.5%)	8 (8.8%)	17 (5.8%)	0.313
Alcoholic abuse	7 (1.8%)	4 (4.4%)	3 (0.9%)	0.036
Body mass index †				0.037
Normal	293 (76.3%)	65 (71.4%)	239 (72.6%)	
Overweight and obese	91 (23.7%)	26 (28.6%)	54 (16.4%)	
Fever	173 (45.1%)	46 (50.5%)	127 (43.3%)	0.228
Gastrointestinal symptoms	126 (32.8%)	34 (37.4%)	92 (31.4%)	0.290
White blood cell (×10^9^/L)	3.55 (2.83)	3.48 (1.36)	3.59 (2.11)	0.384
Lymphocytes (×10^9^/L)	1.04 (0.95)	1.09 (0.97)	1.04 (0.94)	0.860
C-reactive protein (mg/L)	10.4 (4.9)	14.2 (10.8)	9.3 (4.5)	< 0.001
Erythrocyte sedimentation rate (mm/h)	18 (12)	21 (17)	17 (10)	< 0.001
AST(U/L)	24 (15)	63 (55)	19 (13)	< 0.001
ALT(U/L)	24 (16)	68 (62)	21 (13)	< 0.001
ALP(U/L)	60 (49)	67 (51)	58 (49)	0.004
GGT(U/L)	19 (13)	31 (26)	16 (12)	< 0.001
Total bilirubin(μmol/L)	13.1 (9.7)	13.1 (10.5)	13.1 (9.2)	0.002
Direct bilirubin(μmol/L)	2.6 (1.8)	3.5 (2.4)	2.4 (1.6)	< 0.001
Indirect bilirubin(μmol/L)	10.2 (7.3)	9.9 (7.8)	10.0 (7.1)	0.025
D-dimer (mg/L)	0.62 (0.49)	0.63 (0.52)	0.59 (0.49)	0.242
Prothrombin time (s)	13.9 (13.1)	13.8 (13.2)	13.9 (13.1)	0.384
Activated partial thromboplastin time (s)	31.4 (28.5)	32.1 (30.1)	30.9 (28.4)	0.154
Type of COVID-19				< 0.001
Mild	168 (43.8%)	40 (44.0%)	128 (43.7%)	
Commen	208 (54.2%)	43 (47.3%)	165 (56.3%)	
Severe	7 (1.8%)	7 (7.7%)	0 (0.0%)	
Critical	1 (0.3%)	1 (1.1%)	0 (0.0%)	
Chinese traditional medicine treatment	341 (88.8%)	79 (86.8%)	262 (89.4%)	0.491
Lian-Hua-Qing-Wen	341 (88.8%)	79 (86.8%)	262 (89.4%)	0.491
Qing-Fei-Pai-Du-Tang	288 (75.0%)	76 (83.5%)	212 (72.4%)	0.032
Xuan-Fei-Bai-Du-Tang	27 (7.0%)	10 (11.0%)	17 (5.8%)	0.091
Shen-Qi-Shi-Yi-Wei	14 (3.6%)	38 (41.8%)	52 (17.7%)	0.556
Qiang-Li-Pi-Pa	33 (8.6%)	8 (8.8%)	25 (8.5%)	0.939
Su-Huang-Zhi-Ke	23 (6.0%)	6 (6.6%)	17 (5.8%)	0.781
Ling-Yang-Jiao	55 (14.3%)	16 (17.6%)	39 (13.3%)	0.310
Huo-Xiang-Zheng-Qi	71 (18.5%)	23 (25.3%)	48 (16.4%)	0.056
Others	85 (22.1%)	22 (24.2%)	63 (21.5%)	0.591
Acetaminophen treatment	126 (32.8%)	33 (36.3%)	93 (31.7%)	0.422
Low molecular weight heparin treatment	285 (74.2%)	69 (75.8%)	216 (73.7%)	0.689
COVID-19 neutralizing antibody treatment	11 (2.9%)	5 (5.5%)	6 (2.0%)	0.085
Mechanical ventilation	8 (2.1%)	8 (8.8%)	0 (0.0%)	< 0.001
ICU admission	8 (2.1%)	8 (8.8%)	0 (0.0%)	< 0.001
Death	1 (0.3%)	1 (1.1%)	0 (0.0%)	0.072
Length of stay (days)	14 (12)	15 (12)	14 (12)	0.072

* *P* value of the comparison between the liver injury group and the control group. † Body mass index = Weight(kg) / Height(m)^2^. Normal, 18.5 kg/m^2^ < body mass index < 24 kg/m^2^; Overweight, 24.0 kg/m^2^ ≤ body mass index < 28.0 kg/m^2^ ; Obese, body mass index ≥ 28 kg/m^2^. Abbreviations: COVID-19: 2019 novel coronavirus disease; aspartate aminotransferase, AST; alanine aminotransferase, ALT; alkaline phosphatase, ALP; γ-Glutamyl transferase, GGT; intensive care unit, ICU.

## Abbreviations

COVID-19: 2019 novel coronavirus disease
ICU: intensive care unit
ALT: alanine aminotransferase
AST: aspartate aminotransferase
ALP: alkaline phosphatase
GGT: γ-Glutamyl transferase
